# Supplementary material for: Assessing the genetic diversity of Ethiopian indigenous goat ecotypes at the hemoglobin locus and its associations with morphometric traits
Source: PLoS One. 2025 Aug 29;20(8):e0330451. doi: 10.1371/journal.pone.0330451 (PMC12396699; doi:10.1371/journal.pone.0330451)
Supplement: S1 Table — (DOCX) [file pone.0330451.s001.docx]

**S 1 Table. The interaction effects hemoglobin genotype and agroecological zone on morphometric traits.**

| Genotype *agroecological zone | BW | CG | HW | BL | RL | RW | RH | CD | FCC | EL |
| --- | --- | --- | --- | --- | --- | --- | --- | --- | --- | --- |
| Midland AA | 27.1±0.9 | 70.3±1.1 | 66.0±0.8 | 65.5±0.8 | 20.5±0.3 | 14.4±0.3 | 66.0±0.7 | 30.4^ab^±0.4 | 8.0±0.1 | 13.6±0.2 |
| Midland AB | 28.6±0.6 | 71.9+0.7 | 67.6±0.5 | 65.1±0.5 | 20.4±0.2 | 14.9±0.2 | 67.6±0.5 | 31.1^a^±0.3 | 8.0±0.1 | 13.8±0.2 |
| Midland BB | 35.8+1.4 | 76.7+1.7 | 71.5±1.2 | 67.3±1.3 | 22.3±0.5 | 15.7±0.5 | 70.8±1.2 | 31.4^ab^±0.6 | 8.4±0.2 | 14.4±0.4 |
| Highland AA | 25.8±0.7 | 71.8+0.9 | 63.2±0.6 | 64.3±0.6 | 19.3±0.3 | 13.9±0.2 | 64.1±0.6 | 30.6^ab^±0.3 | 7.7±0.1 | 12.7±0.2 |
| Highland AB | 25.3±0.8 | 70.2+0.9 | 63.6±0.7 | 62.1±0.7 | 19.6±0.3 | 13.9±0.3 | 64.1±0.7 | 30.5^ab^±0.4 | 7.7±0.1 | 12.5±0.2 |
| Highland BB | 30.8±1.1 | 75.0+1.3 | 64.7±0.9 | 62.8±0.9 | 21.3±0.4 | 14.0±0.4 | 65.2±0.9 | 29.2^b^±0.5 | 7.6±0.2 | 12.8±0.3 |
| Lowland AA | 29.6±0.9 | 74.1+1.1 | 68.4±0.8 | 65.6±0.8 | 21.0±0.3 | 15.4±0.3 | 67.4±0.8 | 29.3^b^±0.4 | 8.2±0.1 | 14.9±0.2 |
| Lowland AB | 29.9±0.7 | 73.6+0.9 | 68.4±0.6 | 65.7±0.6 | 20.9±0.3 | 15.4±0.2 | 66.8±0.6 | 29.9^ab^±0.3 | 8.0±0.1 | 14.8±0.2 |
| Lowland BB | 34.2±0.9 | 77.6+1.0 | 71.5±0.7 | 65.8±0.8 | 22.3±0.3 | 15.4±0.3 | 70.2±0.7 | 31.0^ab^±0.4 | 8.5±0.1 | 15.0±0.2 |
| P - value | 0.3011 | 0.437 | 0.240 | 0.249 | 0.817 | 0.593 | 0.152 | 0.0036 | 0.188 | 0.580 |

a,b,ab= values under the column are significantly different; BW= body weight; CG= chest girth; HW= height at withers; BL=body length; RL= rump length; RW= rump width; RH= rump height; CD= chest depth; FCC= fore canon circumference; EL= ear length
