# Supplementary material for: Assessing the genetic diversity of Ethiopian indigenous goat ecotypes at the hemoglobin locus and its associations with morphometric traits
Source: PLoS One. 2025 Aug 29;20(8):e0330451. doi: 10.1371/journal.pone.0330451 (PMC12396699; doi:10.1371/journal.pone.0330451)
Supplement: Attachment — Submitted filename: Response to Reviewers.docx [file pone.0330451.s008.docx]

**May 12, 2025**

**Manuscript Title: *Assessing the genetic diversity of Ethiopian indigenous goat ecotypes at the hemoglobin locus and its associations with morphometric traits***

**Dear editors,**

The authors have addressed all the comments and suggestions provided by reviewers and the academic editor. Both reviewers and the academic editor are acknowledged for their time and feedback, which contributed to the improvement of the manuscript. Revisions have been made throughout the manuscript from abstract to references. Specifically, the abstract has been refined for clarity and readability. The introduction has been revised and updated with recent literature. The methodology section has been revised according to the reviewers' recommendations. Additional results, supported by newly added figures, have been included. The discussion section has been updated to address the reviewers' comments comprehensively, and approximately 33 new references have been incorporated and cited. Furthermore, data and supporting information have been provided as supplementary files. All changes made in the manuscript are highlighted in blue for ease of reference.

| S/n | **RESPONSE TO REVIEWER #1** | | | |  |
| --- | --- | --- | --- | --- | --- |
|  | **Comments given** | | | **Responses** |  |
| 1 | Revise the introduction to explicitly connect the findings to broader goals, such as improving productivity or resilience in goat populations. | | | The introduction was revised based on the provided comment. It now elaborates on genetic diversity, emphasizing its essential role in driving genetic improvement and facilitating adaptation. Moreover, it highlights the importance of Hb polymorphisms in examining genetic diversity, adaptation and productivity, and underscores their potential as markers for selecting animals in genetic improvement programs. |  |
| 2 | Clarify and simplify the abstract for improved readability | | | The abstract has been improved to make it easier to understand, especially in **lines 31-34, 36-37 and 39-42.** |  |
| 3 | Revise the introduction to highlight the study’s practical relevance in breeding programs and livestock productivity. | | | Thank you for this valuable comment. The role of Hb polymorphisms in livestock breeding program and genetic improvement was highlighted in the introduction especially from lines **62-83**. |  |
| 4 | Practical challenges of implementing genotype-based selection in smallholder farming systems, where resources may be limited and potential solutions | | | The challenges of genotype-based selection under a smallholder farming system and **potential solutions** were presented in detail (**lines 437-462)** |  |
| 5 | Provide additional context on deviations from HWE and heterozygosity results compared to global benchmarks | | | The deviation from HWE and its context from different perspective, including relevant scientific assumptions, with comparisons to global benchmarks, are provided in **lines 364-383.** |  |
| 6 | Strengthen language and grammar throughout the manuscript | | | The language and grammars throughout the manuscript have been thoroughly revised and improved. |  |
| 7 | Write name of equation in line 180 and methods in line 181. | | | Thank you for your suggestion. The names of equations and methods are now provided in **lines 179 and 180.** |  |
| 8 | Include visual aids (graphs, charts) to summarize key results. | | | **Thank you very much for your suggestion**.  1. The distribution pattern of total Hb is presented in a pie chart and provided as supplementary material (**S1 Fig.**)  2. The distribution of Hb genotypes based on agroecology is shown in a chart (**S2 Fig.**). (S1 and S2 Figs) were provided as supplementary files. However, further summarization of results through visual aids was not feasible. For example, variables such as heterozygosity, genetic distance, and association of morphometric traits with genotype data are not suitable to display in graphical form. |  |
| **No** | | **RESPONSE TO REVIEWER #2** | | | |
|  | | **The given comments** | **Responses** | | |
| **1** | | The sample size (225 goats) may not be sufficiently large to capture the full extent of genetic variability within Ethiopian indigenous goat populations, potentially limiting the generalizability of the findings. | Thank you for this important observation. While a sample size of 225 goats may appear limited when considering the total population of Ethiopian goats estimated at over 46 million heads and comprising more than 14 distinct breeds. Our study focused on southeastern Ethiopia. We purposefully selected goats from three representative districts across three agro ecological zones: Highland, midland, and lowland. This approach ensured the inclusion of variations in management practices, nutritional status, and production systems. Given this targeted design, we believe the sample is appropriate for assessing genetic diversity at the Hb locus and its association with key performance traits within the study area. Moreover, many peer- reviewed studies published in international journals have utilized comparable or smaller sample sizes when examining genetic markers in livestock. To support the adequacy of our sample, we have listed some aricles with their sample size and doi that provides examples of such studies. These references demonstrate that our sample size is consistent with accepted scientific practice, particularly in studies focused on local breeds and targeted loci such as hemoglobin variants. Therefore, we are confident that our findings provide meaningful insights, particularly for the regions and contexts studied. **Articles** \| **Sample size** \| **Doi.** \| **Species**; Gwaza et al., 2019 \| 40 \| https://doi.org/10.18488/journal.57.2019.81.38.47 \| Goat; Agaviezor et al.,2013 \| 60 \| https://doi.org/10.13140/2.1.1552.2246 \| Goat; Osaiyuwu & Salako, 2018 \| 100 \| https://doi.org/10.5897/ajb2015.14655 \| sheep; Važić et al., 2017 \| 189 \| https://doi.org/10.2298/GENSR1701151V \| Sheep; Orunmuyi et al., 2020 \| 155 \| https://doi.org/10.1007/s11250-020-02257-y \| Poultry; Chebo et al., 2024 \| 284 \| https://doi.org/10.1007/s11250-024-04086-9 \| Poultry; Pal and Mummed, 2014 \| 105 \| https://doi.org/10.14202/vetworld.2014.229-233 \| Cattle | | |
| **2** | | Second, while the study identifies associations between hemoglobin polymorphisms and morphometric traits, it does not establish causal relationships, and other genetic or environmental factors influencing these traits were not explored. | Thank you for this valuable observation. While our study focuses on association rather than causation, there are several biological reasons to explore the link between the Hb variant and morphometric traits and other traits related to production, disease resistance, and adaptability. Additionally, there was budget limitation to explore other genetics and environmental factors. Hemoglobin is a complex protein composed of two α-globin and two β-globin subunits, which are encoded by separate genes. These subunits influence the oxygen-biding capacity and transport efficiency, which are critical for cellular metabolism and overall physiological growth. Variations in Hb structure can lead to differences in oxygen delivery to tissues, potentially affecting growth rate, body size, and other morphometric traits, especially under varying environmental conditions. Some Hb variants also have a selective advantage against disease and increase the survivability of animals. Additional context and explanation regarding these mechanisms have been provided in the discussion part (**lines 317- 328**). | | |
| **3** | | **Third**, the study focuses solely on the hemoglobin locus, whereas multiple genes contribute to morphometric traits and adaptability, limiting a comprehensive genetic assessment. | We acknowledge that production and morphometric traits are polygenic, influenced by multiple genes with varying effects (e.g., QTLs). However, the Hb locus is known to be highly polymorphic across species and may play a significant role in productivity and adaptability. These effects could arise through mechanisms such as dominance, pleiotropic and epistasis, and interactions with other locus and general heterozygosity. Due to its polymorphic nature and physiological importance, we believe the Hb locus contributes meaningfully to the expression of performance and adaptive traits.  # Additionally **we recommended** the integration of broader genomic tools such **as genetic mapping and microarray-based technologies to further examine gene expression and identify genes affecting a wider range of traits.**  The role of hemoglobin in adaptability and performance traits is discussed in the manuscript (**lines 66-77 and 420-435).** | | |
| **4** | | **Fourth,** although significant differences were observed between agro-ecological zones, the study does not account for management practices, nutritional status, or selective breeding histories, which could influence the results. | Since the study was carried out under on-farm conditions, agro ecological variation inherently includes differences in nutrition, management practices, feed and water availability, and overall husbandry systems. These factors are integral components of agrological variation and are important for evaluating the actual genetic potential, adaptability, and performance of animals in their natural production environments. By considering these real-world variations, our study aims to ensure that future genetic improvement programs are aligned with the practical conditions in which the animals are raised.  To further strengthen the result we have provided detailed information on:   1. The management of experimental animals in the methodology section (**lines 122-131**); 2. The possible reasons for performance differences among the three agroecologies, from the local study context and broader global insights, in the discussion section (**lines 270-277** and **280-286**).   The interaction between Hb genotype and agroecology, with the results presented in the results section **(lines 255-260)** and summarized in Supplementary Table 1 (**S1 Table**). | | |
| **5** | | **Fifth**, functional validation through molecular or physiological studies was not conducted to confirm the potential role of Hb polymorphisms in adaptation or productivity. | Thank you for raising this important point. Our study did not include functional validation through molecular or physiological experiments to directly confirm the role of Hb polymorphisms in adaptation or productivity. This is due to lack of budget to undertake other molecular and physiological study. The allocated budget is only for hemoglobin polymorphisms study (for blood sample collection and analysis). However, our research was designed as an exploratory genetic and phenotypic association study under field conditions, aiming to identify potential links between Hb variants and traits of interest in diverse agroecologies. The functional significance of hemoglobin polymorphisms has been widely discussed in the scientific literature, and we have incorporated relevant findings to support our interpretations. These are articulated in the introduction **(from line 66-74**) and further discussed in the discussion section (**lines 319-325)**. | | |
| **6** | | **Finally**, the deviation from Hardy-Weinberg Equilibrium (HWE) in midland goats suggests possible selection, mutation, migration, or non-random mating, but these factors were not further investigated. Future studies should incorporate larger sample sizes, whole-genome approaches, and environmental variables to provide a more comprehensive understanding of the genetic diversity and adaptive potential of indigenous goats. | Thank you for this valuable comment. Indeed, deviations from Hardy-Weinberg Equilibrium (HWE) are common in natural populations, particularly under smallholder farming systems where ideal HWE assumptions, such as random mating, no selection, mutation, migration, or genetic drift are rarely met. In our study, we discussed the possible explanations for the observed deviation in midland goats, including natural and artificial selection, gene flow through migration (for feed or market purposes), and small effective population sizes. These points are elaborated in the manuscript (**lines 370-383)** within local and broader contexts.  While we acknowledge that the current study did not investigate each of these evolutionary forces in detail due to its scope and design, we agree with the reviewer that future research should employ larger sample sizes, incorporate whole-genome approaches, and consider environmental and management variables to explore the genetic architecture and adaptive potential of indigenous goat populations. These recommendations have been incorporated in the conclusion section. | | |
| **7** | | **Apart from these technical evaluations**, another important point concerns the **capacity** of the data. The study may be highly valuable at the local level. However, its contribution to goat breeding or goat genetics on an international scale is quite limited. Local breeds are crucial, and their genetic characterization provides valuable data for biodiversity conservation. However, the international applicability of such datasets and their adaptation to larger populations are quite challenging. I believe it would be more appropriate to publish these findings in regional or specialized journals that focus on local livestock genetics and biodiversity. | We fully acknowledge the importance of aligning scientific contributions with local relevance and broader international applicability. Hemoglobin polymorphisms are of global scientific interest due to their well-documented polymorphic nature, simple pattern of inheritance, and their association with key performance and adaptive traits including, productivity, disease resistance, adaptability, and fertility. As demonstrated in our study and the literature, Hb variants have functional relevance and are valuable genetic markers in selective breeding programs. In addition, Hb genotyping is low-cost, accessible, and easy to interpret, making it a practical tool for many developing countries where advanced genomic technologies may not be widely available. We believe this study provides insights that go beyond local application. Although focused on southeastern Ethiopia, the methodology, findings, and implications regarding Hb variation and its phenotypic associations are relevant for researchers, breeders, and policymakers working with indigenous livestock populations under low-input systems globally. Most importantly, the integration of Hb genotypes with morphometric traits enhances the broader significance of the work by linking molecular markers with observable performance characteristics. We appreciate the suggestion regarding publication venue, but respectfully submit that our study addresses pressing questions in livestock genetics and adaptation that are also of international relevance, particularly in the context of biodiversity conservation and climate-resilient breeding strategies. | | |

| **No** | **RESPONSE TO ACADEMIC EDITOR** | |
| --- | --- | --- |
|  | **The given comments** | **Responses** |
| **1** | Please ensure that your manuscript meets PLOS ONE's style requirements, including those for file naming. | Thank you for the reminder. The manuscript has been formatted according to the Plos One Author guidelines, including file naming conventions. |
| **2** | PLOS requires an ORCID iD for the corresponding author in Editorial Manager on papers submitted after December 6th, 2016. Please ensure that you have an ORCID iD and that it is validated in Editorial Manager. | The corresponding author’s existing ORCID iD has been validated in Editorial Manager. |
| **3** | In your Methods section, please provide additional details regarding participant consent from the owners of the animals. In the ethics statement in the Methods and online submission information, please ensure that you have specified (1) whether consent was informed and (2) what type you obtained (for instance, written or verbal). If the need for consent was waived by the ethics committee, please include this information. | Thank you for the suggestion. Participant consent has been included in the methods section (**Lines 133-139)**. Initially, some farmers were hesitant expressing concerns that blood sample collection might harm their animals by causing weight loss or exposing them to diseases. To address this the researchers reassured the farmers that all blood samples would be collected by trained professionals with veterinary expertise and that the procedure would not harm the animals. An informal oral agreement was reached with each participating farmer. Following this mutual understanding, the blood and morphometric data were collected.  The ethical standard of the research was reviewed and approved by the Research Ethics Review Committee (RERC) of Hawassa University. This information is provided in the methodology section under Ethics approval (**Lines 95-97**). |
| **4** | We note that the grant information you provided in the ‘Funding Information’ and ‘Financial Disclosure’ sections do not match. When you resubmit, please ensure that you provide the correct grant numbers for the awards you received for your study in the ‘Funding Information’ section. | Thank you for pointing this out. **We have now provided the correct grant number and included the names of the award recipients in the “**Funding Information” section. |
| **5** | In the online submission form, you indicated that the data will be made available upon reasonable request. All PLOS journals now require all data underlying the findings described in their manuscript to be freely available to other researchers, either 1. In a public repository, 2. Within the manuscript itself, or 3. Uploaded as supplementary information. This policy applies to all data except where public deposition would breach compliance with the protocol approved by your research ethics board. If your data cannot be made publicly available for ethical or legal reasons (e.g., public availability would compromise patient privacy), please explain your reasons on resubmission and your exemption request will be escalated for approval. | Thank you for the clarification. All relevant data including raw data, SAS and pop gene outputs were added as **supplementary information** at the end of the manuscript after the reference list. |
