# Supplementary material for: Assessing the genetic diversity of Ethiopian indigenous goat ecotypes at the hemoglobin locus and its associations with morphometric traits
Source: PLoS One. 2025 Aug 29;20(8):e0330451. doi: 10.1371/journal.pone.0330451 (PMC12396699; doi:10.1371/journal.pone.0330451)
Supplement: Attachment — Submitted filename: Plose HB.docx [file pone.0330451.s007.docx]

Title: **Assessing the genetic diversity of Ethiopian indigenous goat ecotypes at the hemoglobin locus and its associations with morphometric traits**

**General comment,**

The manuscript presents a valuable concept by focusing on the understanding and documentation of genetic diversity in goats using protein markers. The authors have effectively addressed the concerns raised by the previous reviewers, resulting in a clearer and more readable manuscript. However, the manuscript will benefit if the authors consider the following minor comments.

Line 28: Relevant morphometric traits …. Please state the traits

Line 29: The red cells were subjected … The red blood cells were subjected…

Line 39 - : In conclusion, the Hb polymorphisms could serve as a genetic marker for selective breeding programs to enhance productivity and adaptability of indigenous goats.

- Do you have any evidence that which ones are adaptive/productive and which ones are not? If yes, please state it in one sentence

Line 41: A genome-wide association study is recommended to validate the association of Hb variants with economically important traits.

- This is wrong and should be deleted

Line 69: … exhibit greater tolerance under unfavorable conditions than … what unfavourable condition – state the condition

Line 117: … digital portable weighing balance having a 50kg capacity. State the precision of the weighing scale

Line 146: Hemoglobin electrophoresis

- State how blood was collected, transported and stored before going to lab analysis.

Line 185 … The statistical model used to test the relationships between hemoglobin genotypes and morphometric variables was as follows:

Yij = μ+Ai +Hj+ e

- This is a GLM model to check the effect of HB type on performance, not a correlation model – you need to have this correlation model if you want the association

Line 210, S1 and S2. This is an important information and is the main result - need to be presented in the main document.

Line 437: Challenges of genotype-based selection under smallholder farming

- I do not understand the importance of this section – please remove it.
