# Supplementary material for: Assessing the genetic diversity of Ethiopian indigenous goat ecotypes at the hemoglobin locus and its associations with morphometric traits
Source: PLoS One. 2025 Aug 29;20(8):e0330451. doi: 10.1371/journal.pone.0330451 (PMC12396699; doi:10.1371/journal.pone.0330451)
Supplement: Attachment — Submitted filename: Responses to reviwers.docx [file pone.0330451.s009.docx]

**RESPONSES TO REVIEWERS	July, 25/2025**

**Manuscript titles: Assessing the genetic diversity of Ethiopian indigenous goat ecotypes at the hemoglobin locus and its associations with morphometric traits**

**Dear editor,**

The authors have addressed all the comments and suggestions provided by reviewers. We would like to express our sincere appreciation to all esteemed reviewers for taking the time to critically review the manuscript, which helped us to improve the manuscript. Revisions have been made on different sections of the manuscript as recommended by reviewers. About four (4) new references have been included to address the reviewer's comment in the introduction and methods section and properly cited in the references list. In addition, about fifteen (15) unnecessary references have been removed with their underlying ideas while addressing the comments. Moreover, we have reviewed all the references under the reference list to check for completeness and retraction using the “Retraction Watch Database” and have ensured that there is no retracted paper cited in this manuscript. For the sake of clarity and convenience, any modification made to the document has been highlighted with track changes mode. All the reviewer comments and the author’s responses have been provided in the table form below.

**RESPONSE TO REVIEWR #3**

| **No.** | **Section** | **Reviewer’s Comments** | **Authors responses** |
| --- | --- | --- | --- |
| **1** | **Introduction** | Lines 120-124: Here it is stated that there are no studies on Hb in Ethiopia. However, a brief search revealed the following two studies. Therefore, these studies should be evaluated in this section and necessary citations should be made. Simachew A., Bekele E., 2013. Genetic variatıon of some goat populations in Ethiopia by means of blood protein polymorphism. Ethiop. J. Biol. Sci. 12(2): 169-186,  Simachew, Addis, 2002. Characterization of some Goat Populations in Ethiopia by means of Blood Protein Polymorphism. Thessis. <https://hdl.handle.net/10568/67965> | Thank you for the suggestions. We have evaluated and cited these articles appropriately. After careful examination of these two works, they address the same topic, similar sample size, study area, methods, and results. This is because the article published is the version of the thesis, and therefore, we have cited the recent one (2013). We have highlighted this study in the introduction section (Lines 88-95). |
| **2** | **Materials and Methods** |  |  |
| **1** |  | No information was given on the age of the goats used in this study. Were goats of the same age or different ages used as experimental animals? Considering that morphometric characteristics were also determined in the study, ages are important. Because morphometric characteristics will be affected by age. | We have sampled adult goats with similar age groups (4PPI), i.e., four years and above. The age was estimated from their dentition as proposed by Ebert and Solaiman (2010). This information has been stated (lines 120-122 and 127-129). |
| **2** |  | Additional information on the morphometric characteristics to be obtained from the experimental animals is required. In addition, relevant references should be cited to explain the methodology by which these characteristics will be determined. | We have collected some morphometric traits from the experimental goats to evaluate the effect of Hb genotype on those traits. The morphometric traits were measured by following the FAO guidelines for phenotypic description of animals (FAO, 2012). |
| **3** |  | It is stated that POPGENE32 program was used in the data analysis section. First, this program should be cited as indicated below. Secondly, all of the calculations mentioned in lines **176-191** are made with the help of this program. Therefore, there is no need to write equations or give details about the calculations. | The POPGENE32 has been properly cited as recommended. Yeh FC, and Boyle TJB. (1997). Population genetic analysis of co-dominant and dominant markers and quantitative traits. Belgian Journal of Botany 129: 157. All the formulas that indicate manual calculation have been deleted as recommended lines(177-179). |
|  | **Results** | **In Table 2** the number of genotype considering both sex and the number of genotype in the overall total is incompatible. | After reanalyzing the sex data using the pop gene, we verified all of the errors and made the necessary corrections as indicated by the lines (225–227) and in Table 2. Additionally, fig. 1 shows the total genotype frequency. Pop gene output for the effects of sex and agroecological zone is available as supplemental materials files S2 and S3 Txt. |

**RESPONSES TO REVIEWER 4#**

| **No.** | **Section** | **Reviewer’s comments** | **Authors responses** |
| --- | --- | --- | --- |
| **1** | **Abstract** |  |  |
|  |  | Line 28: Relevant morphometric traits …. Please state the traits | **Thank you for suggestions**. The collected morphometric traits have been included (Lines 27-29) |
|  |  | Line 29: The red cells were subjected … The red blood cells were subjected | Done (Line 29) |
|  |  | Line 39: In conclusion, the Hb polymorphisms could serve as a genetic marker for selective breeding programs to enhance productivity and adaptability of indigenous goats.  Do you have any evidence that which ones are adaptive/productive and which ones are not? If yes, please state it in one sentence | There are different reports regarding the role of Hb in the adaptation and productivity of animals. It has been reported that Hb^AA^ is mostly known for its high oxygen affinity and is found in a higher proportion of animals in high-altitude areas and is resistant to helminth infection. At the same time, there are also reports that indicate the role of this genotype in productivity traits. Whereas the Hb^BB^ is known for its high water content, low viscosity, and abundance in animals living in lowland arid areas. In addition, there are different reports regarding the role of Hb variants in productivity traits. However, to explicitly state/articulate which one is adaptive and which one is productive, further study may be required. Generally, it’s difficult to generalize and specifically ascertain and articulate the role of each genotype independently based on the existing data. However, we can say generally hemoglobin has a role in adaptation and production. Therefore, we have revised the statement. |
|  |  | Line 41: A genome-wide association study is recommended to validate the association of Hb variants with economically important traits. - **This is wrong and should be deleted** | This sentence has been removed as recommended. |
| **2** | **INTRODUCTION** |  |  |
|  |  | Line 69: … exhibit greater tolerance under unfavorable conditions than … what unfavorable condition – state the condition. | The hemoglobin Hb^AA^ genotypes have a selective advantage in high-altitude areas due to their high oxygen affinity, higher Hb concentration, and packed cell volume. This feature allows them to live in higher altitudes, unfavorable feed, and resistance to helminth infections. Therefore, the unfavorable conditions low temperature (cold area), oxygen-deficient conditions, and helminth infections (line 68). |
|  | **Materials and methods** |  |  |
|  |  | Line 117: … digital portable weighing balance having a 50kg capacity. State the precision of the weighing scale | We have been stated (line 127-129). The digital portable scale with high accuracy and a precision of 10 g was utilized to measure live body weight. |
|  |  | Line 146: Hemoglobin electrophoresis - State how blood was collected, transported and stored before going to lab analysis. | The blood sample collection procedures and storage and transport system have been presented (Lines 152-158). |
|  |  | Line 185 … The statistical model:  Yij=μ+Ai+Hj+e This is a GLM model to check the effect of HB type on performance, not a correlation model–you need to have this correlation model if you want the association | We have used this model to test the effect of hemoglobin genotypes on morphometric traits. Morphometric data were collected from similar animals in which a blood sample was collected. The blood samples and morphometric data were labeled with similar numbers to indicate which linear data is collected from which animals. Then the Hb genotypes were inserted in Excel, which contains morphometric data. For example, goat 1HF is a female animal sampled from highland agroecological zone. The genotype and morphometric measurement of the 1HF goat are filled directly on the Excel sheet. That is the case we call association. Moreover, the Hb genotypes are categorical (AA, AB, and BB), and their values cannot be expressed in numbers. Thus, while associating them with morphometric traits, it’s difficult to use a correlation model. Instead, we have used the PROC GLM model by considering the Hb genotypes as a fixed effect. |
|  | **RESULTS** |  |  |
|  |  | Line 210, S1 and S2. This is an important information and is the main result - need to be presented in the main document. | Both figures have been presented in the main document (Fig 1 and Fig 2). |
|  | **DISCUSSION** | Line 437: Challenges of genotype-based selection under smallholder farming. **I do not understand the importance of this section–please remove it.** | We have removed this part. |

**RESPOSES TO REVIWER 5#**

| **No.** | **Reviewer’s comment** | **Authors responses** |
| --- | --- | --- |
|  | The authors need to provide the gel picture of electrophoresis and incorporate it in the manuscript. | The gel pictures have been provided as supplementary materials (S1 **Fig**). |

**RESPONSES TO EDITOR**

| **No.** | **Editor’s comments** | **Authors responses** |
| --- | --- | --- |
| **1** | Please review your reference list to ensure that it is complete and correct. If you have cited papers that have been retracted, please include the rationale for doing so in the manuscript text, or remove these references and replace them with relevant current references. Any changes to the reference list should be mentioned in the rebuttal letter that accompanies your revised manuscript. If you need to cite a retracted article, indicate the article’s retracted status in the References list and also include a citation and full reference for the retraction notice. | We have reviewed, edited, and corrected all references in the reference list. We have also ensured all references under the citation are properly cited in the reference list. We have checked the retracted paper using the “**Retraction Watch Database**” by inserting the author’s name, title, journal, and DOI. Based on the results of the Retraction Watch Database, all references used in the manuscript are not retracted, or no reference is found in retraction status. In addition, we have edited the comma, semicolon, and journal names to make them consistent and in Vancouver style. Moreover, based on the reviewer’s comments, we have included four (4) new references and removed/deleted about fifteen (15) references with the underlined data. |
